# Prevalence and Molecular Identification of Nematode and Dipteran Parasites in an Australian Alpine Grasshopper (*Kosciuscola tristis*)

**DOI:** 10.1371/journal.pone.0121685

**Published:** 2015-04-28

**Authors:** Kate D. L. Umbers, Lachlan J. Byatt, Nichola J. Hill, Remo J. Bartolini, Grant C. Hose, Marie E. Herberstein, Michelle L Power

**Affiliations:** 1 Department of Biological Sciences, Macquarie University, North Ryde, New South Wales, Australia; 2 School of Biological Sciences, University of Wollongong, Wollongong, New South Wales, Australia; 3 Centre for Evolutionary Biology, School of Animal Biology, University of Western Australia, Crawley, Western Australia, Australia; 4 School of Science and Health, University of Western Sydney, Sydney, New South Wales, Australia; Federal University of Viçosa, BRAZIL

## Abstract

In alpine Australia, Orthoptera are abundant, dominant herbivores, important prey species, and hosts for parasites and parasitoids. Despite the central role of orthopterans in alpine ecosystems, the impact of parasites on orthopteran populations is under-explored. In this study we describe the relationship between parasite prevalence and host sex, body size and year of collection. We accessed an existing, preserved collection of 640 *Kosciuscola tristis* collected from across its range between 2007 and 2011. Upon dissection we collected juvenile parasites and used molecular tools to identify them to three families (Nematoda; Mermithidae, and Arthropoda: Diptera: Tachinidae and Sarcophagidae). The prevalence of nematodes ranged from 3.5% to 25.0% and dipterans from 2.4% to 20.0%. Contrary to predictions, we found no associations between parasite prevalence and grasshopper sex or size. Although there was an association between prevalence of both nematodes and dipterans with year of collection, this is likely driven by a small sample size in the first year. Our results provide a foundation for future studies into parasite prevalence within the alpine environment and the abiotic factors that might influence these associations.

## Introduction

Parasites are ubiquitous and can have profound impacts on host immunology, morphology, behavior and fitness. Host-parasite interactions built on single-host and single-parasite systems under laboratory conditions are becoming well understood, [1–4] but data on the impacts of parasite infection on host fitness in the field are scarce. A major obstacle in understanding the interactions between parasites and their hosts is the lack of basic knowledge of the prevalence and diversity of parasites. This is particularly true for Australia and crucial for understanding threatened ecosystems such as the Australian alpine region.

Many alpine insect species are highly abundant throughout the summer and occur in high densities in resource rich areas [5]. Such distributions can enhance parasite transmission due to increased contact with infected individuals [6] and greater susceptibility to infection arising from the elevated stress of living under crowded conditions [7]. For example, monarch butterflies (*Danaus plexippus*) reared at high densities have elevated stress responses and greater susceptibility to parasitism than those in low-density populations [8]. Given that alpine environments are not only harsh but stochastic, it is common that conditions vary greatly from year to year [9]. The combination of high population densities and non-trivial environmental stressors may facilitate the increased parasitism reported in a range of alpine insects [10–12].

Orthoptera are well-known as parasite vectors [13–16] and there is some evidence to expect that host sex and size are related to prevalence. Zuk and McKean [17] summarise the relationship between parasite susceptibility in males and testosterone indicating that across taxa so far studied males have a higher susceptibility to parasites and carry higher parasite loads. For example, Miura and Ohsaki [18] showed that host-parasite interactions may be driving sexual size dimorphism in a Japanese catantopine grasshopper where larger females have fewer parasites.

Australia’s alpine region harbors a rich assemblage of grasshoppers [5,19] and toward the end of Summer and into Autumn these dominant herbivores are very common. During their seasonal peak (March to May) and above 1500 m altitude *Kosciuscola tristis* (Acrididae: Oxyinae) is found in high density across its range and exhibits unusual attributes for a grasshopper such as fierce fighting in males [20–23] and temperature-dependent colour change [20,24]. In laboratory colonies, adult *K*. *tristis* expelled unidentified worm-like parasites, larvae and pupae and these observations prompted this study. Thus, we investigated parasites of *K*. *tristis* to quantify and qualify infection in this dominant alpine species. Specifically, we aimed to: (1) identify parasites using molecular tools, (2) examine variation in parasite prevalence associated with year, sex and body.

## Materials and Methods

### Collection and dissection

Between 2007 and 2011 we established a preserved collection of *K*. *tristis* (n = 640) for genetic studies. Grasshoppers were collected in Kosciuszko National Park, NSW, Australia along Dead Horse Gap Walking Track and Merritt’s Nature Trail out of Thredbo Village (36°30′S 148°18′E) and the ‘Blue Calf T-Bar’ out of Guthega Village (36°23′S 148°22′E) (NSW National Parks and Wildlife Scientific License S12256). These three collection sites harbor members of a single interbreeding population [25,26]. Grasshoppers were caught by hand and identified using a field key [5] and via the presence of a conical prosternal process [27]. Sex was determined by gross morphology, genitalia and colouration. Grasshoppers were cooled over night in a refrigerator and then stored in 70% ethanol for transport to Macquarie University by car.

Prior to dissection and as a proxy for body size, the dorsal midline of the pronotum (representing the narrowest length) was measured the using digital calipers (0–150 mm, Sontax Australia, Perth, Australia). Grasshoppers were then bisected along the medial line of the venter and the exoskeleton splayed to reveal the internal abdomen, thorax and head. Samples were systematically searched for parasites (Nematoda and Diptera). Dissections were performed using a stereo microscope (Olympus SZ40, Olympus, Japan), parasites were preserved in 70% EtOH until DNA extraction and remaining grasshopper parts useful for DNA extraction were returned to 70% EtOH.

### Molecular identification of parasites

Parasite identification was confirmed by sequencing the 18S rDNA. Total DNA was extracted from a midsection of nematodes (1 cm sample sectioned aseptically with scalpel blade) and whole or half dipteran larvae using the Qiagen DNeasy tissue kit (Qiagen Australia). The manufacturers recommended protocol for DNA extraction from insect material was followed and included the following minor modifications. Samples were ground using a micropestle in a 1.5 mL Eppendorf tube before and after proteinaseK digestion, and an additional wash step was included during precipitation. Amplification of the 18S rDNA locus was initially achieved using the universal eukaryotic primers EukF- AACCTGGTTGATCCTGCCAG and EukR- CCTTCTGCAGGTTCACCTAC to obtain full-length 18S rDNA sequences for representative samples (DeLong 1992). Sequences generated from EukF and EukR amplicons were then used to develop taxa specific primers for subsequent analyses of all samples using a hemi-nested design. For screening nematode samples the secondary reaction included the primers EukF and nem595R- CGAAAAATCAACTACGGGCG, and the secondary primers for dipteran amplification were EukF and tach693R —ACCGGTAATACGCTTRCATACA. All reactions were performed using GoTaq Green Master Mix (Promega, Madison, USA) with the cycling conditions for EukF and EukR encompassing an initial denaturation of 3 min at 94°C; 35 cycles at 94°C for 30 s, 56°C for 30 s and 72° C for 2 min; and a final extension step at 72°C for 5 min. For secondary reactions the annealing temperature was increased to 60°C and primary product (1 μL) was used as DNA template.

Reactions were performed in an Eppendorf Mastercycler (Eppendorf, North Ryde, Australia). Amplicons were visualized by agarose gel electrophoresis (2% *w/v*) in TBE (TRIS, boric acid, EDTA; pH 8) with SYBR safe staining (Invitrogen, Mulgrave, Australia). Amplicon size was approximated against a HyperLadderII DNA marker (Bioline, Sydney, Australia). Amplicons were purified using the QIAquick PCR Purification Kit (Qiagen, Melbourne, Australia) and sequenced in the forward and reverse directions. Sequencing was performed on a 3130x1 genetic analyzer (Applied Biosystems, Foster City, California) using the BigDye terminator kit (Applied Biosystems) and the standard run protocol for a 50 cm, 16 capillary array.

### Sequencing and Phylogenetic analyses

**Fig 1:**
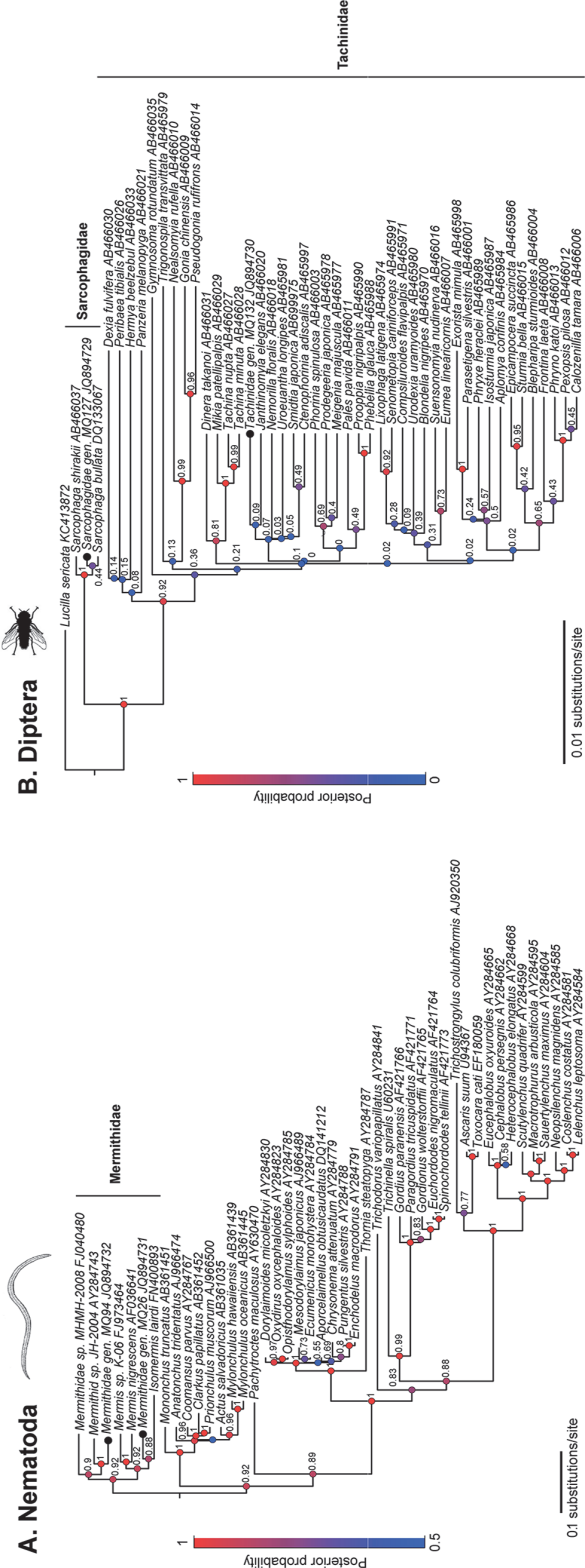
Phylogenetic trees of the 18s rDNA gene analysed using Bayesian inference (BEAST) places the Nematoda isolates within the family Mermithidae and the Diptera isolates within two families, the Tachinidae and Sarcophagidae. **Isolates from this study are indicated by a black circle (●).** The Diptera tree (A) included 48 sequences and a total of 646 nucleotide positions. The Nematoda tree (B) included 46 sequences and a total of 1644 nucleotide positions. Posterior probabilities indicate statistical support at each branch node and are colour coded according to level of support (1: high, 0:low)

Sequences of 18S rDNA were manually checked for read error and then assembled into optimized contigs using Geneious (version 5.5.6, Bioinformatics software Biomatters Ltd, New Zealand). Contigs covered 1409 and 655 nucleotides for the Nematoda and Diptera isolates, respectively. A BlastN search (www.ncbi.gov) including only sequences with >95% query coverage was performed to assign contigs to the lowest resolution taxonomic group. Sequences generated from this study were supplemented with publicly available sequences representing major lineages of Nematoda and Diptera (accession numbers given in Fig 1A & 1B) and aligned using ClustalW with default parameters [28]. The nucleotide sequences identified in this study were submitted to GenBank under the accession numbers (JQ894729—JQ894732).

Phylogenetic analysis was performed using Bayesian Markov Chain Monte Carlo method implemented in BEAST v1.8.0 [29]. For all analyses the random local molecular clock and the General Time Reversible substitution model accounting for estimates of invariable sites (I) and the gamma distribution parameter (G) were used. The analysis was conducted using a Bayesian skyride coalescent tree prior. We performed two independent analyses of 20 million generations that were combined after appropriate burnin (~10%) to produce 10,000 trees. Branch placement within the phylogenetic tree was used to assign isolates from this study to family level.

### Correlates of parasite prevalence in Kosciuscola tristis

We tested whether prevalence of infection varied according to host sex, host body size, year of collection and stage of development. Of the 640 grasshoppers dissected, 103 were subadult and as such we could not confidently determine their sex. Thus, for our analysis of the effect of sex on prevalence we excluded subadult animals and used 385 male and 152 female adults (total n = 537). We used a Generalized Linear Model (link function = logit) with binomial error structure. Analyses were performed using the GLM function in the stats module of R version 2.14.0 (R Core Development Team 2011). Presence of parasite was the dependent variable with host sex, host body size, year of collection and stage of development as independent variables each tested separately. Analyses of Deviance were based on the chi-square test. We analyzed nematode and dipteran infection separately.

## Results

### Molecular identification of parasites

We successfully amplified the 18S region for all 50 nematodes. For the 64 putative dipteran larvae collected, 50 18S sequences (from 49 grasshopper hosts) were confirmed Diptera, the remaining 14 were either host tissue mistaken for small larvae (4 samples) or failed extractions or amplifications likely due to extremely small amounts of starting tissue in those samples. A BlastN search of these isolates showed > 95% sequence similarity to either Nematoda or Diptera genera. Representative sequences of Nematoda; MQ26 and MQ94 and Diptera; MQ127 and MQ132 were selected for phylogenetic analyses. Bayesian analysis placed the two Nematoda isolates (MQ26 and MQ94) within the Mermithidae family (Fig 1A). Nematoda samples clustered into two genetically distinct clades with 93.2% pairwise identity to each other. Both isolates showed highest identity (MQ26: 93.25%; MQ94: 96.00%) with *Mermithidae* sp. isolate JH-2004 (AY284743). Previous morphological studies suggest *Mermithidae* isolates in Australian grasshoppers may be related to *M*. *quirindeansis* or *M*. *athysanota* [30,31]. For the dipteran sequences, Bayesian analysis identified MQ127 as belonging to the Sarcophagidae flesh flies with 100% similarity to *Sarcophaga bullata* (DQ133067) (Fig 1B). In contrast, isolate MQ132 clustered within the Tachinidae (Fig 1B), a large and speciose family of parasitoid flies. MQ132 showed highest identity (99.23%) with *Smidtia japonica* (AB699975) and *Senometopia cariniforceps* (AB465991).

### Patterns of parasite prevalence in *K*. *tristis*

**Fig 2:**
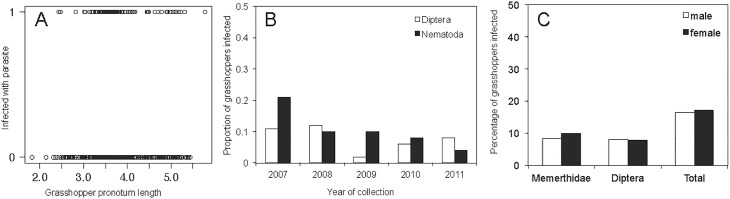
Patterns of parasite presence in *Kosciscola tristis*: (A) Relationship between grasshopper body size and whether or not they were infected. (B) Prevalence of Nematoda and Diptera in *K*. *tristis* over five years ranged between 3.5% to 21.4%. The overall prevalence of Nematoda and Diptera in *K*. *tristis* (n = 640) was 7.8% and 7.7%, respectively, (C) Proportion of males and females infected with Nematoda and Diptera.

The prevalence of Nematoda in *K*. *tristis* was 7.8% overall and ranged between 3.5% and 25.0% over the five-year study period (Fig 2; Table 1). When present, only a single nematode was found in any individual grasshopper. Of the two genetic variants identified by 18S rDNA sequencing, 36 of the 50 infected grasshoppers, were infected with Genus 1 (MQ94) and 14 with Genus 2 (MQ26) (Genus 1–2007: 4, 2008: 7, 2009: 7, 2010: 11, 2011: 7; Genus 2–2007: 2, 2008: 9, 2009: 3, 2010: 0, 2011: 0). There was a significant difference in the prevalence of infection by nematodes between years of collection with a peak in prevalence observed in 2007 (Fig 2), however this is most likely an effect of low sample size in 2007 (n = 28) and that these samples were collected at the very end of the season (May) (Table 2). Pooling all years, we found no significant difference in the prevalence of nematode infection with host size, sex or stage of development (Tables 1 & 2 and Fig 2).

**Table 1:** Numbers of grasshoppers that were infected with Diptera and/or Nematoda parasites from 2007 to 2011.

Year	Total N *K*. *tristis*	Overall Diptera prevalence (%)	Overall Nematoda Prevalence (%)	N females	Female pronotum mm±SD	N females with Diptera (%)	N females with Nematoda (%)	N males	Male pronotum mm±SD	N males with Diptera (%)	N males with Nematoda (%)	N infected subadults (Diptera, Nematoda)
2007	28	3 (11)	6 (21)	15	4.76±0.27	2 (13)	5 (33)	12	3.55±0.28	1 (8)	1 (8)	1 (0)
2008	169	21 (12)	16 (10)	40	4.89±0.45	7 (18)	0 (0)	129	3.66±0.30	14 (11)	16 (12)	0 (0)
2009	101	1 (2)	10 (10)	47	4.07±0.89	0 (0)	7 (15)	52	3.56±0.23	1 (2)	3 (6)	0 (0)
2010	145	9 (6)	11[12] (8)	30	4.51±0.33	1 (3)	1 (3)	115	3.72±0.28	8 (7)	10 (9)	0 (0)
2011	199	15 (8)	7 (4)	20	4.83±0.47	2 (10)	1 (2)	77	3.42±0.29	7 (9)	2 (3)	10 (6, 4)
**Total**	**640**	**49 (8)**	**50 (8)**	**152**	**4.61±0.34**	**12 (8)**	**14 (9)**	**385**	**3.58±0.12**	**32 (8)**	**32 (8)**	**11 (3)**

Overall numbers of infected individuals are presented, as well as numbers of females and males infected and their pronotum length (proxy for body size). Final row provides total counts for prevalence and averages for pronotum sizes.

**Table 2:** Generalized Linear Model results of ecological correlates of infection by dipteran and nematode parasites.

**Nematoda infection**	df	Wald—χ^2^	P
Host sex	1	0.32	0.57
Host body size	1	0.06	0.81
Male host body size	1	0.13	0.72
Female host body size	1	1.34	0.24
Year of collection	4	12.51	**0.01**
Stage of development	1	3.29	0.07
**Diptera infection**	df	Wald—χ^2^	P
Host sex	1	0.01	0.95
Host body size	1	0.01	0.93
Male host body size	1	4.08	**0.04**
Female host body size	1	2.20	0.14
Year of collection	4	15.32	**<0.01**
Stage of development	1	0.62	0.43
**Combined infection**	df	Wald—χ^2^	P
Host sex	1	0.15	0.70
Host body size	1	0.01	0.93
Male host body size	1	1.51	0.22
Female host body size	1	0.06	0.80
Year of collection	4	12.97	0.11
Stage of development	1	3.60	0.06

The overall prevalence of Diptera ranged between 1.9% and 12.4% from 2007 to 2011, inclusive (Fig 2; Table 1). The majority of Diptera were from Tachinidae, only three individual Sarcophagidae were present. Similarly to Nematoda, there was a significant difference in the prevalence of infection by Diptera in different years (p < 0.01, Table 2; Tachinidae—2007: 2, 2008: 21, 2009: 1, 2010: 9, 2011: 14, from 2007 to 2011; Sacrophagidae—2007: 1, 2008: 0, 2009: 0, 2010: 0, 2011: 2; one grasshopper was infected with two Diptera, each of a different family). It seems that individual hosts can be infected with multiple larvae at once. Our initial visual inspection of the presence of Diptera larvae in individual *K*. *tristis* hosts varied between one and three with one larva found in 35 grasshoppers, two larvae in 13 grasshoppers and three larvae in one grasshopper, however, our molecular identification could not confirm this in all but one case where the same host was infected by Diptera from two families. Pooling data across years, there was no significant relationship between prevalence of Diptera infection and size of all grasshoppers, or of males and females separately (Table 2). There was also no significant difference in the prevalence of Diptera infection between the stage of development however we did see a difference between the sexes where male body size varied with infection but female body size did not (Tables 1 & 2). When combining Diptera and Nematoda infection we found no relationships between infection and any of the parameters included in the analysis (Table 2).

## Discussion

### Molecular approach to parasite identification

We describe molecular identification of parasites from nematode and dipteran in *K*. *tristis*, an Australian alpine grasshopper. While morphology enables identification to phylum, molecular characterisation of isolates enabled finer-scale classification within phylum (Nematoda) and order (Diptera) to family level namely, Mermithidae, Sarcophagidae and Tachnididae. This is the first molecular analysis of these taxa in the Australian environment. Previous identifications of Mermithidae, Sarcophagidae and Tachindae in insect hosts in Australia have been based on morphological identifications [32–34]. The morphological approach can be limiting because characters used to identify both nematodes and dipterans are predominantly found in adult life stages [10,34]. By applying molecular tools we identified juvenile parasite life-stages in preserved host material without having to rear them to final instar [35–37].

### Parasite prevalence and life history

The overall prevalence of mermithids (Nematoda) in *K*. *tristis* is similar to prevalence reported in other insect hosts [15,38–40]. Belonging to a group of water loving grasshoppers (Oxyinae) [27], the association between *K*. *tristis* and mermithid parasites may not be surprising given the preference of mermithids for moist soil conditions [41]. The mermithid transmission pathway in *K*. *tristis* is not known but may include direct or indirect modes of transmission as in other herbivorous insects [42,43]. The effects of mermithids on grasshopper hosts include mortality [44] and reduced fecundity [15]. In *K*. *tristis* we have observed that grasshoppers survive mermithid emergence when held in captivity for days to weeks suggesting that mortality is not at least not an immediate outcome of mermithid emergence. Nevertheless, the presence of a mermithid parasite may impact one or more of several life history traits including, vulnerability to predation, microhabitat choice, fecundity and genital morphology [45–48]

Phylogenetic inference placed *K*. *tristis*’ parasitic dipterans in two families: Tachinidae and Sarcophagidae. These families are both known insect parasites and representative species have been identified in other Australian orthopteran hosts (e.g. Morabinae grasshoppers) [32,33]. Of the two families of Diptera we identified, the Sarcophagidae were less prevalent (found in three individual grasshoppers) while tachinids were common (found in 47 grasshoppers) (Table 1). Adult tachinids are common in alpine and snowfall regions of Australia and have also been found in approximately half the grasshopper populations studied in New Zealand, USA and Sweden [10,40,49]. We have observed tachinid pupae emerging from grasshoppers during high-temperature experimental treatments (>35°C) (from which the grasshoppers survived) but we have not systematically collected data on emergence cues. Point of infection and persistence of tachinid larvae in hosts would be of interest.

### Characteristics of hosts and parasite prevalence

The pattern of parasite prevalence in *K*. *tristis* was varied among the three variables we measured for this study (host sex, host body size and year of collection). Although statistical differences are likely driven by a small sample size in 2007, our data show that parasite prevalence fluctuates from year to year but with no clear increase or decline over the five years studied (Fig 2). Fluctuations in parasite prevalence from 2007 to 2011 may be a result of spatial and temporal heterogeneity of the alpine environment, but further monitoring is required to confirm this.

We found no relationship between parasite prevalence and host body size for nematodes, but we did for male body size and dipterans (Fig 2). This may indicate that the relationship between host size and parasite infection is species specific among Orthoptera. For example, in *Parapodisima tanbaensis* Miura and Ohsaki [18] found that larger females were less likely to be parasitised. However, in two species of grasshopper inhabiting tallgrass prairie, Laws and Joern (2012) showed that larger females were more likely to be infected than smaller females but a relationship between female size variability and parasite prevalence was not evident for an additional seven species studied. Similarly, in our study, grasshopper female body size showed no relationship with infection. Comparative studies across grasshoppers would provide a better perspective for understanding the complexities of host parasite energetics and its impact on body size [50].

Finally, our data show no clear patterns between host sex and prevalence for either parasite phylum (Fig 2). This was surprising as accounts of male sex-biased parasitism are numerous [17,51]. For example, mate attraction behaviour may drive prevalence differences between the sexes [52] as in the tachinid parasite *Ormia ochracea*, which is attracted to the call of male crickets [53]. Although some studies have reported sex-biased parasitism in invertebrate hosts [54,55], our data are consistent with findings that sex biased parasitism in many arthropod hosts is lacking [56].

The host-parasite system that we describe in this study has an alpine distribution, the host is a superabundant herbivore, an important prey item, and is endemic to the Australian alpine region. The data contributed here enhance our understanding of the ecology of *Kosciuscola* grasshoppers and their parasites and provide a foundation for examining the field ecology of this interaction.

## Supporting Information

S1 Database Table containing data on grasshoppers collected for this study and their parasite load. (TXT)
